# gB co‐immunization with GP96 enhances pulmonary‐resident CD8 T cells and exerts a long‐term defence against MCMV pneumonitis

**DOI:** 10.1111/jcmm.16065

**Published:** 2020-11-06

**Authors:** Bingnan Guo, Peifeng Xu, Dafei Chai, Lei Cao, Lin Liu, Tengfei Song, Shuqun Hu, Yuling Chen, Xianliang Yan, Tie Xu

**Affiliations:** ^1^ Jiangsu Institute of Health Emergency Xuzhou Medical University Xuzhou China; ^2^ Emergency Center The Affiliated Hospital of Xuzhou Medical University Xuzhou China; ^3^ Department of Respiratory Medicine The Affiliated Hospital of Xuzhou Medical University Xuzhou China; ^4^ Cancer Institute Xuzhou Medical University Xuzhou China; ^5^ The Feinstein Institute for Medical Research Manhasset, New York NY USA; ^6^ Department of Emergency Nanjing Jiangning Hospital Nanjing China

**Keywords:** cytomegalovirus, DNA vaccine, GP96, pneumonitis, pulmonary‐resident CD8 T cell

## Abstract

Human cytomegalovirus (HCMV) infection in the respiratory tract leads to pneumonitis in immunocompromised hosts without available vaccine. Considering cytomegalovirus (CMV) mainly invades through the respiratory tract, CMV‐specific pulmonary mucosal vaccine development that provides a long‐lasting protection against CMV challenge gains our attention. In this study, N‐terminal domain of GP96 (GP96‐NT) was used as a mucosal adjuvant to enhance the induction of pulmonary‐resident CD8 T cells elicited by MCMV glycoprotein B (gB) vaccine. Mice were intranasally co‐immunized with 50 μg pgB and equal amount of pGP96‐NT vaccine 4 times at 2‐week intervals, and then *i.n*. challenged with MCMV at 16 weeks after the last immunization. Compared with pgB immunization alone, co‐immunization with pgB/pGP96‐NT enhanced a long‐lasting protection against MCMV pneumonitis by significantly improved pneumonitis pathology, enhanced bodyweight, reduced viral burdens and increased survival rate. Moreover, the increased CD8 T cells were observed in lung but not spleen from pgB/pGP96‐NT co‐immunized mice. The increments of pulmonary CD8 T cells might be mainly due to non‐circulating pulmonary‐resident CD8 T‐cell subset expansion but not circulating CD8 T‐cell populations that home to inflammation site upon MCMV challenge. Finally, the deterioration of MCMV pneumonitis by depletion of pulmonary site‐specific CD8 T cells in mice that were pgB/pGP96‐NT co‐immunization might be a clue to interpret the non‐circulating pulmonary‐resident CD8 T subset expansion. These data might uncover a promising long‐lasting prophylactic vaccine strategy against MCMV‐induced pneumonitis.

## 1 INTRODUCTION

As a double‐stranded DNA virus, cytomegalovirus (CMV) belongs to the β‐herpesvirus subfamily.^1^ Human cytomegalovirus (HCMV) infection in the respiratory tract results in pneumonitis in the immune deficiency host, which often goes along with a severe mortality.^2^ Currently, little efficient vaccines or therapeutic reagents are available against HCMV infection—especially for HCMV pneumonitis.^3^ Due to strict species specificity of CMV and the similarities between murine cytomegalovirus (MCMV) and HCMV in virion structure, gene expression, tissue tropism and latency, mouse infected by MCMV is the most widely used model to simulate human HCMV infection.^2^, ^4^

Vaccination is an attractive avenue to prophylaxis against infection diseases. So far, there are only three therapeutic vaccines that have completed phase II clinical trials against CMV infection.^5^ All three vaccines focus on an important envelope glycoprotein B (gB), including two with a neutralizing antibody and one DNA vaccine.^6^, ^7^, ^8^ However, in CMV‐seronegative woman, the proportion of protection was only 50% by gB/MF59 vaccine.^7^ gB plasmid alone could only provide partial protection (62.5%) over 4 times immunization in a mouse model setting.^9^ Therefore, new vaccination strategies and potent adjuvants should be developed to improve a long‐lasting protection efficacy.

Mucosal immunization might provide a way for protection against CMV pneumonitis and prevent the spread of virus.^10^ Mucosal DNA vaccines were potent to control diseases by the induction of specific T‐cell immune responses in lung.^11^ Studies had shown that appropriate mucosal immunization strategy had been successfully applied to control MCMV infection^12^, ^13^, ^14^; however, compared with other approaches, the induction of immune tolerance but not activation was often associated with mucosal immunization because of its limited immunogenicity.^15^ Hence, a success of mucosal vaccine should employ appropriate and effective adjuvant to enhance the immunogenicity. Heat‐shock proteins (HSPs), which are produced by cells under stress condition, had been well characterized as initiator and stimulator of immune responses to fight against infectious diseases.^16^ GP96, which played an important role in the cross‐presentation of antigens to MHC I and MHC II molecules, had been known to activate antigen‐specific T‐cell responses.^17^ Moreover, previous reports showed that N‐terminal domain of GP96 (GP96‐NT) had an adjuvant function in virus‐specific CTL immune responses.^18^ Thus, GP96‐NT might be beneficial to the potential protection effects of gB vaccine against MCMV pneumonitis.

Memory T cells provided a long‐term protection against pathogens which had been eliminated. Once been re‐exposure to the same pathogen, memory T cells could quickly convert to effector T cells and proliferate robustly to fight against the pathogen.^19^ Recently, a distinct memory CD8 T subset (CD8 T_RM_) that resided in non‐lymphoid tissues such as lung, gut, skin, brain or kidney, was CD69^+^ and CD103^+^, had no capacity of recirculation. However, it could offer a rapid and robust protection against pathogens and tumours at the local site.^20^ CMV infection induced broadly functional T‐cell responses, which generated a stronger long‐term memory response compared to other pathogens.^21^ Specific CD8 T cells that targeted CMV were accumulated in tissues and maintained at high levels during CMV infection.^22^, ^23^ These reports provided a theoretical basis that a suitable vaccination strategy could offer a long‐lasting protection against MCMV pneumonitis.

This study aims to determine whether MCMV gB plasmid combined with GP96‐NT plasmid could provide a long‐term protection against MCMV pneumonitis. We demonstrated that pgB/pGP96‐NT plasmids intranasal co‐vaccination could efficiently moderate the severity of MCMV pneumonitis 16 weeks after the last immunization. Protection efficacy went closely along with the enhanced induction of pulmonary but not splenic CD8 T‐cell immune responses. Moreover, pulmonary‐resident CD8 T cells increment in pgB/pGP96‐NT co‐immunization upon MCMV challenge might depend on enhancement of lung CD8 T_RM_ that facilitated pulmonary‐resident CD8 T‐cell expansion. Our study might unlock an novel vaccination strategy for MCMV pneumonitis.

## 2 MATERIALS AND METHODS

### 2.1 Animals and virus

6‐ to 8‐week‐old female Balb/c (H‐2^d^) mice were purchased from experimental animal centre of Chinese Academy of Science. All mice were bred and maintained under pathogen‐free conditions. All animal experimental protocols were approved by the Care and Use of Laboratory Animals (Ministry of Health, China, 1998) and the guidelines of the Laboratory Animal Ethical Committee of Xuzhou Medical University. MCMV (Smith strain) was a gift from Prof. Lizeng Qin (Shandong Academy of Medical Sciences, Shandong, China) and was maintained by passage through NIH 3T3 cells. High virulent salivary gland‐derived MCMV, propagated in Balb/c mice by 10 serial in vivo passages for virulence enhancement, was used in challenge experiments. MCMV stock had a titre on 3T3 cells of 10^8^ PFU/ml and a 50% lethal dose (LD_50_) of approximately 10^6^ PFU virus particles in Balb/c mice. Normal lethal challenge or lethal challenge was performed with 3LD_50_ or 5LD_50_ virus stock.

### 2.2 Preparation and Transfection of plasmids

To generate GP96‐NT gene, the region of nucleotides 1–1,014 of GP96^24^ was amplified from mouse spleen into the plasmid expression vector pcDNA3.1. To generate MCMV, gB (GenBank: M86302.1) was amplified from high virulent MCMV‐infected 3T3 cells into the plasmid expression vector pcDNA3.1. The primers used in this study were listed as follow: GP96‐NT, forward primer: 5′ATGCTAGCATGAGGGTCCTGTGGGTGT3′ (Nhe I site); reverse primer: 5′GCGGATCCATCATTCATAAGTTCC3′(BamH I site). MCMV gB, forward primer: 5′GCCAAGCTTATGTCAAGAAGAAACGAAAGAGGATGT3′ (Hind III site); reverse primer: 5′GGCGGATCCTCAGTACTCGAAATCGGAGTCCTCCGCC3′ (BamH I site).

For cell transfection assays, plasmids were resolved in PBS at a concentration of 1 mg/ml. 5 × 10^5^ 3T3 cells were transfected with 4 μg of pgB or pGP96‐NT in 35‐mm dishes by PEI (Sigma) according to the manufacturer's protocol. Same amount of empty vector pcDNA3.1 was used as a negative control. 48 hours after transfection, cells were harvested for further experiments.

### 2.3 Western blot

Cells or tissues were lysed in cold lysis buffer containing 20 mmol/L Tris‐HCl pH7.4, 150 mmol/L NaCl, 1 mmol/L EDTA and 0.5% NP‐40. Cell debris was depleted by centrifugation at 6000 g for 5 min. Equal amount of protein was loaded to 12% SDS‐PAGE gel for each sample, separated by electrophoresis and transferred to PVDF membrane. The membrane was probed with anti‐mouse GP96 (Novus Biologicals, dilution 1:1000), mouse anti‐gB antibody (Capri, dilution 1:1000) or anti‐β‐actin (CST, dilution 1:1000) followed by goat anti‐mouse/rabbit IgG‐HRP (Southern Biotech, dilution 1:5000). The signals were developed with the SuperSignal West Pico Chemiluminescent Substrate (Thermo Scientific). β‐Actin was used as the internal marker for loading control.

### 2.4 DNA vaccine immunization

pcDNA3.1‐gB (pgB), pcDNA3.1‐GP96‐NT (pGP96‐NT) or pcDNA3.1 was mixed with PEI, respectively, according to the manufacturer's protocol. Mice were divided into four groups: pgB/pGP96‐NT co‐immunization group, pgB immunization group, pGP96‐NT immunization group and pcDNA3.1 (mock vaccine) immunization group. Groups of mice were mildly anaesthetized with pentobarbital (40 mg/kg bodyweight) and intranasally immunized with PEI‐pgB/PEI‐pGP96‐NT, PEI‐pgB, PEI‐pGP96‐NT and PEI‐pcDNA3.1 for 4 times at 2‐week intervals at a dose of 50 μg each plasmid. For pgB alone, pGP96‐NT alone or empty plasmid immunized group, mice received additional 50 μg pcDNA3.1 to ensure that the total DNA amount was 100 μg.

### 2.5 ELISA

For gB‐specific Ab, plates were pre‐coated with 10 μg/ml purified gB protein (Alpha Diagnostic) overnight at 4°C. After blocking with 5% non‐fat milk in PBS, 10‐fold serially diluted serum or alveolar lavage fluid were added in duplicate and incubated for 1 hour. PBS was used as background control, and samples from naive mice were used as negative control. After incubation with HRP‐conjugated goat anti‐mouse IgG and IgA (Southern Biotech) for another 1 hour, TMB substrate (eBioscience) was used to develop colour and absorbance measured at 450 nm. For IL‐2 and IFN‐γ, the BAL fluid was collected at indicated time by using three consecutive instillations of 1 mL PBS at room temperature. The collected BAL fluid was centrifuged at 800 g  at 4°C for 5 minutes, and the supernatants were stored at −80°C for estimation of cytokine levels. IL‐2 and IFN‐γ were measured by ELISA (BioLegend) kits according to the manufacturers' instructions.

### 2.6 MCMV infection and evaluation of pneumonitis

Balb/c mice were infected intranasally with 3LD_50_ of MCMV at week 16 after the final immunization. After 7 days, lung tissues were fixed in 10% phosphate‐buffered formalin, paraffin‐embedded, sectioned and stained with haematoxylin and eosin (HE). The histopathological changes in immunized mice were compared quantitatively by calculating the histopathological scores,^25^ which were scored based on the assignment of one point for each of the following parameters: perivascular inflammation, peribronchial inflammation, interstitial infiltrate or alveolar septal thickening or viral cytopathic effect. Two independent researchers scored separately in a blinded manner. Quantization of viral burden in lung tissues 7 days after 3LD_50_ MCMV challenge, lung tissues were collected, weighed and frozen at −80°C in RPMI 1640 containing 10% FBS. Samples were later thawed, homogenized, serially diluted in 10‐fold increments and incubated on confluent NIH 3T3 cell monolayers for 1 hour at 37°C and 5% CO_2_ to allow viral attachment, and then incubated for 7 days to allow plaque formation. Virus titres were expressed as the mean PFU/100 mg tissue ± SD. For mouse weight loss, mouse bodyweight was measured at the initial day or 7 days after 3LD_50_ MCMV challenge. Mouse survival was monitored daily up to 28 days post‐infection, and Kaplan‐Meier analysis with log‐rank test was used for survival curve.

### 2.7 Isolation of pulmonary hematopoietic cells

Lungs were harvested after perfusion with 10 mL of cold PBS. Tissues were minced using scissors and digested in Hank's balanced salt solution containing 0.2 mg/mL Liberase TM (Roche) and 0.1 mg/mL DNase I (Roche) for 30 min at 37°C. Single‐cell suspension was prepared by passing the tissue through a 70‐μm cell strainer. In some experiments, lymphocytes were further purified using 40% Percoll gradient centrifugation.

### 2.8 labelling of circulating cells and tissue harvest

Mice were *iv* injected with 2.5 μg of anti‐CD45 (A20) PE (BioLegend) in 100 μL of PBS. 3–5 minutes later, mice were killed, and lungs were aseptically isolated within 12 minutes and processed into single‐cell suspensions. The total number of viable cells was determined by trypan blue.

### 2.9 Flow cytometry and cell sorting

Antibodies (Abs) were purchased from BioLegend or eBioscience. Abs used included anti‐CD45(A20), anti‐CD4(RM4‐5), anti‐CD8(53‐6.7), anti‐CD103(2E7), anti‐CD69(H1.2F3), anti‐IFN‐γ(XMG1.2) and anti‐Eomes(W17001A). Intracellular staining of transcription factors was performed using Foxp3 Fix/Perm Kit (Thermo) according to the manufacturer's instructions. For CD4 or CD8 T cells, the plots were pregated on CD45 cells to identify leucocytes firstly; for discriminating circulating or resident CD8 T cells, CD8 T cells were pregated for the further analysis; for pulmonary‐resident CD8 IFN‐γ^+^ analysis, intravenous injection (IV) CD45^‐^ leucocytes were pregated; for lung‐resident CD8 phenotype analysis, IVCD45^‐^CD8^+^ T cells were pregated. Flow cytometric analysis was performed on FACSCanto II (BD Bioscience). Cell sorting was performed using an FACSAria III (BD Biosciences).

### 2.10 T lymphocyte proliferation assay

The sorted pulmonary IV CD45^‐^ CD8 T lymphocytes were labelled with a 5 μmol/L CellTrace CFSE Cell Proliferation Kit (Invitrogen) in PBS with 2% FBS for 20 minutes at 37°C. The labelling reaction was quenched by addition of cold RPMI‐1640 medium with 10% FBS, and cells were washed twice with PBS with 2% FCS to remove excess CFSE. Then, the CFSE‐labelled IV CD45^‐^ CD8 T lymphocytes were added to 96‐well flat‐bottomed tissue culture plates at 5 × 10^5^ cells/well containing 50 U/mL IL‐2 and 200 pfu/mL heat‐inactivation MCMV. The plates were cultured at 37°C with 5% CO_2_ for 3 days. The proliferation of IV CD45^‐^ CD8 T cells was evaluated with CFSE dilution by flow cytometry.

### 2.11 Cytotoxic T lymphocyte (CTL) assays

The sorted pulmonary IV CD45^−^ CD8 T lymphocytes were co‐cultured with 200 pfu/ml heat‐inactivation MCMV for 7 days and used as effector cells. The gB‐transfected autologous SP2/0 cells (H‐2^d^) were used as target cells. Effector cells and target cells were titrated in U‐bottom 96‐well plates at effector‐target cell ratios of 50:1, 25:1 and 12.5:1; thereafter, 1 × 10^4^ target cells were added and incubated at 37°C for 72 h. Cytotoxicity was determined by measuring the amount of lactate dehydrogenase (LDH) in the supernatant with the Cytotoxicity Detection KitPLUS (LDH) (Roche) according to the manufacturer's instructions. To determine the % cell‐mediated cytotoxicity, calculate the average absorbance of the repeated wells subtract the background, then substitute the resulting values in the following equation:

### 2.12 In vivo specific CD8 T‐cell depletion

To specifically deplete mucosal CD8 T cells in the lung, according to published protocol,^26^, ^27^ mice were *i.n*. administered 10 μg αCD8 (clone2.43) or IgG2b (LTF‐2) in 20 μl of PBS −3 day and −1 day following MCMV challenge.

### 2.13 Statistical analysis

Statistical analyses were performed using GraphPad Prism 6 Software (La Jolla, CA). Data were presented as means ± SD or SEM and statistically analysed with two‐tailed independent Student's *t* test (two groups). For mice survival, Kaplan‐Meier analysis with log‐rank test was used. The level of statistical significance was set at *P < .05, **P < .01 and ***P < .001.

## 3 RESULTS

### 3.1 Expression of pgB and pGP96‐NT plasmids in vitro and in vivo

**Figure 1:**
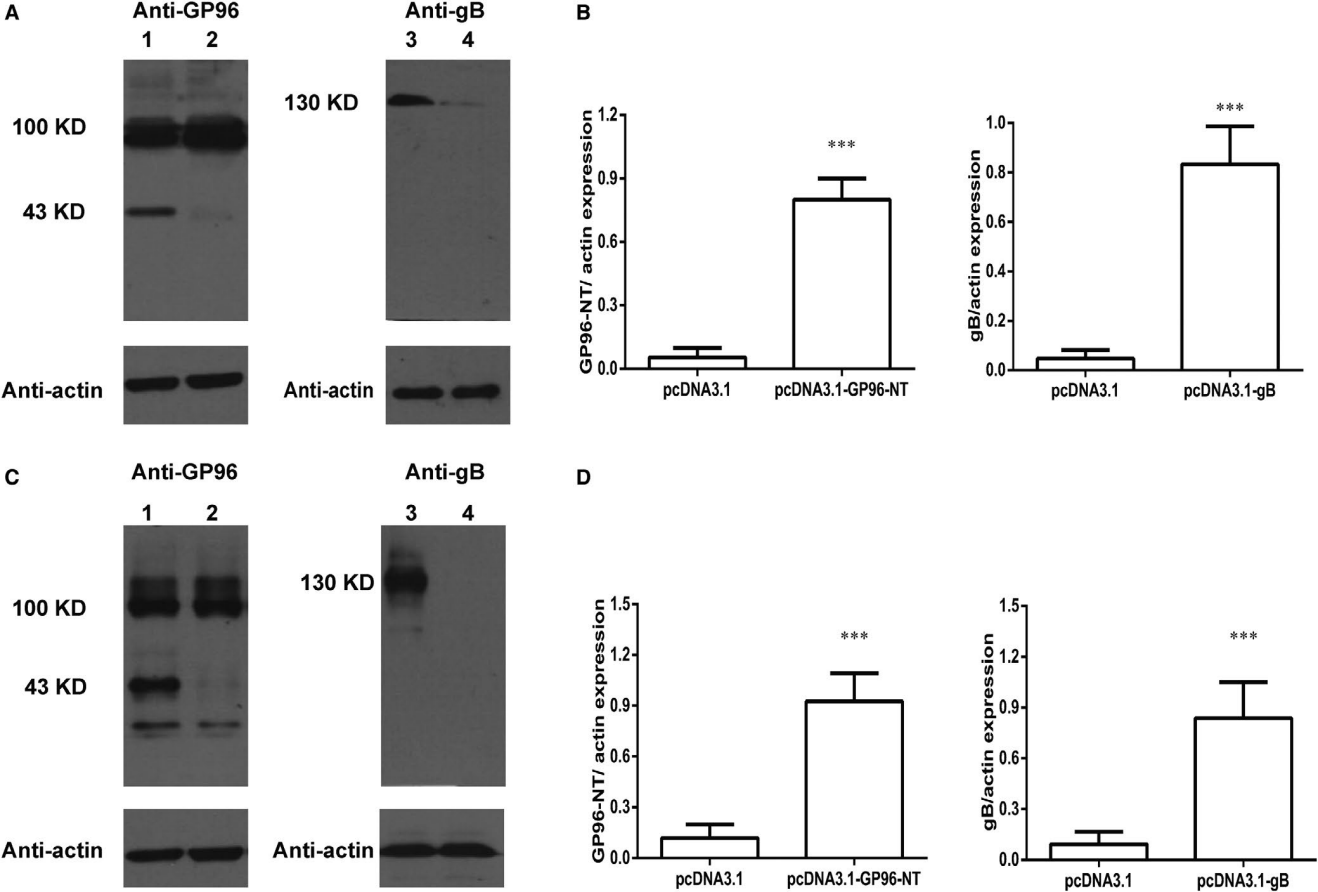
Expression of gB and GP96‐NT plasmids in vitro and in vivo. 3T3 cells were transfected with pgB, pGP96‐NT or vector with PEI for 48 h, and then, cell lysates were subjected to Western blot analysis using anti‐gB, anti‐GP96 or anti‐β‐actin antibody, respectively. A, In vitro expression of gB and GP96‐NT plasmids. Lane 1: 3T3 cells transfected with pcDNA3.1‐GP96‐NT; lane 2: 3T3 cells transfected with pcDNA3.1 vector; lane 3: 3T3 cells transfected with pcDNA3.1‐gB; lane 4: 3T3 cells transfected with pcDNA3.1 vector. B, Quantification of gB and GP96‐NT expression by densitometry in vitro. Data were from one representative experiment of 3 performed and presented as the mean ± SD, and t test was used, ***P < .001. C, Balb/c mice were intranasally immunized with PEI‐pgB or PEI‐pGP96‐NT vaccines, respectively, and lungs were taken 3 days later for gene expression analysis. Western blot analysis of gB, GP96 or β‐actin expression for lung tissues from immunized mice. Lane 1: Mice immunized with pcDNA3.1‐GP96‐NT; lane 2: mice immunized with pcDNA3.1 vector; lane 3: mice immunized with pcDNA3.1‐gB; lane 4: mice immunized with pcDNA3.1 vector. D, Quantification of gB and GP96‐NT expression by densitometry in vivo. Data were from one representative experiment of 3 performed and presented as the mean ± SD (n = 8), and t test was used,***P < .001

To address the expression of pgB and pGP96‐NT plasmid in vitro, 3T3 cells were transfected with 2 plasmids by PEI for 48 h, respectively. The increased expression of gB protein at a size of about 130 KD in pgB‐transfected 3T3 cells was identified by Western blotting assay (Figure 1A,B). Moreover, the expression of GP96‐NT fragment was also detected at the size of 43 KD in relevant transfected cells (Figure 1A,B). As a constitutively expressed protein in mammalian cells, the endogenous expression of full‐length GP96 protein in 3T3 cells was also detected at a molecular weight of 100 KD (Figure 1A). Next, the lungs of mice that received *i.n*. 50 μg pgB, pGP96‐NT or control plasmid were collected for detection of gB or GP96‐NT expression in vivo. Data revealed that in the lungs of pgB‐immunized or pGP96‐NT‐immunized mice, robust expression of gB or GP96‐NT was observed by immunoblotting assay (Figure 1C,D). Taken together, these data confirmed that PEI could serve as an effective vehicle for gene delivery and pgB and pGP96‐NT could be successfully expressed in vitro and in vivo.

### 3.2 Intranasal immunization with pgB/pGP96‐NT boosts adequately immune responses

**Figure 2:**
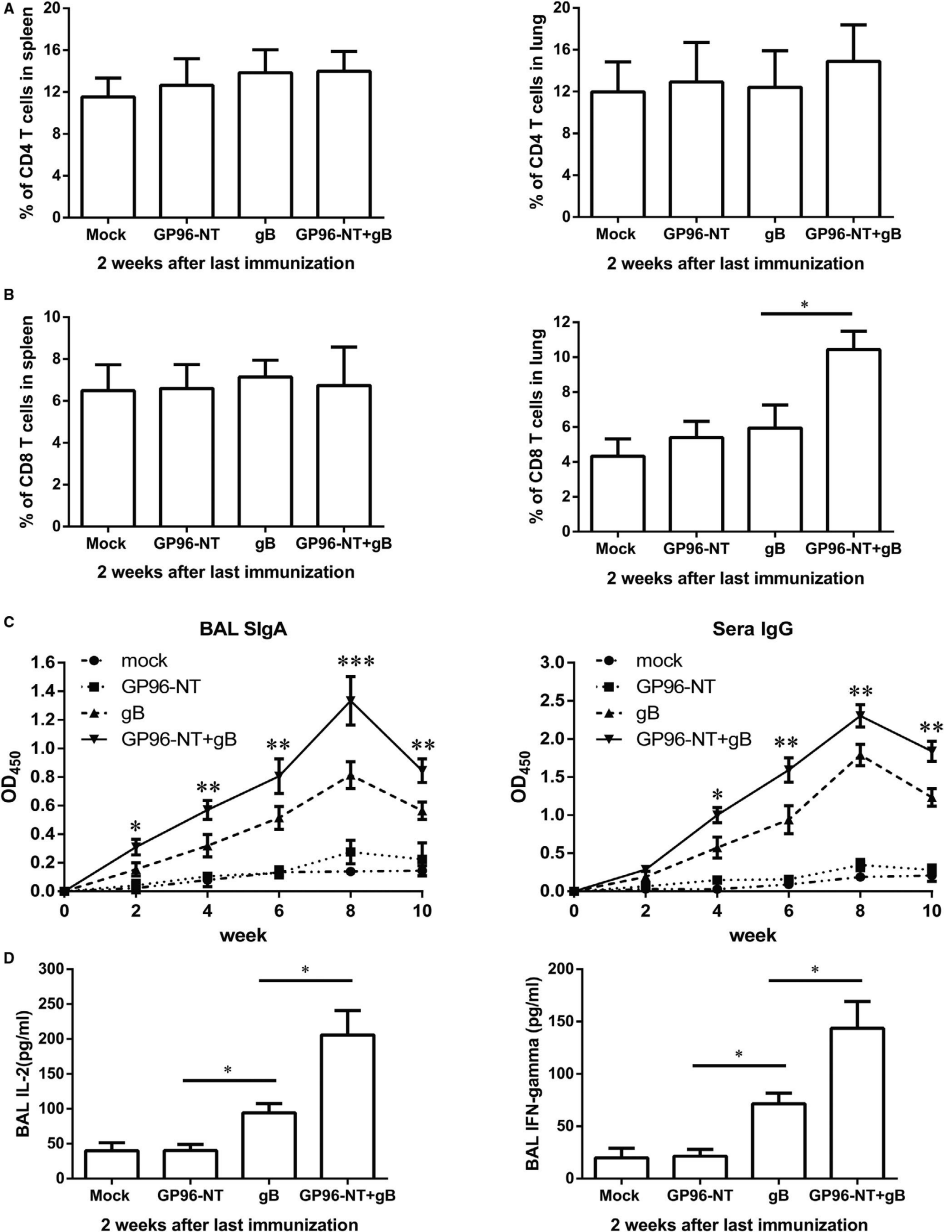
The immune responses are induced by co‐immunization of PEI‐pgB and PEI‐pGP96‐NT. A, B, Frequency of CD4 and CD8 T cells in lung and spleen at week 2 after the last immunization without MCMV challenge. C, Levels of gB‐specific bronchoalveolar lavage fluid SIgA and serum IgG were detected 2 weeks after each immunization without MCMV challenge by ELISA assays. D, ELISA analysis of bronchoalveolar lavage fluid IL‐2 and IFN‐γ at week 2 after the last immunization. Data were from one representative experiment of 3 performed and presented as the mean ± SD (n = 5), and t test was used, *P < .05, **P < .01***P < .001

To address whether intranasal immunization could induce adequately immune responses. Firstly, we evaluated the capacity of intranasal immunization to induce T‐cell responses in both spleen and lung, we found that Balb/c mice were intranasally immunized with 4 doses of vaccines biweekly, 2 weeks after the last immunization, pulmonary but not splenic CD8 T cells had significantly increased by pgB/pGP96‐NT vaccines (Figure 2B). However, CD4 T cells showed no change in both lung and spleen by pgB/pGP96‐NT vaccines (Figure 2A). Besides, gB‐specific BAL SIgA and serum IgG levels robustly induced by pgB/pGP96‐NT or pgB vaccines, which increased gradually after each immunization, with the highest response observed 2 weeks after the last immunization (Figure 2C). Moreover, BAL IL‐2 and IFN‐γ were also significantly increased 2 weeks after the last immunization (Figure 2D). However, both antibodies and cytokines in pgB/pGP96 vaccination group were significantly higher than that in pgB alone group (Figure 2C,D). These results above showed that intranasal immunization route might offer adequately quality and quantity of immune responses.

### 3.3 Intranasal immunization with pgB/pGP96‐NT provides a long‐lasting protection against MCMV pneumonitis in mice

**Figure 3:**
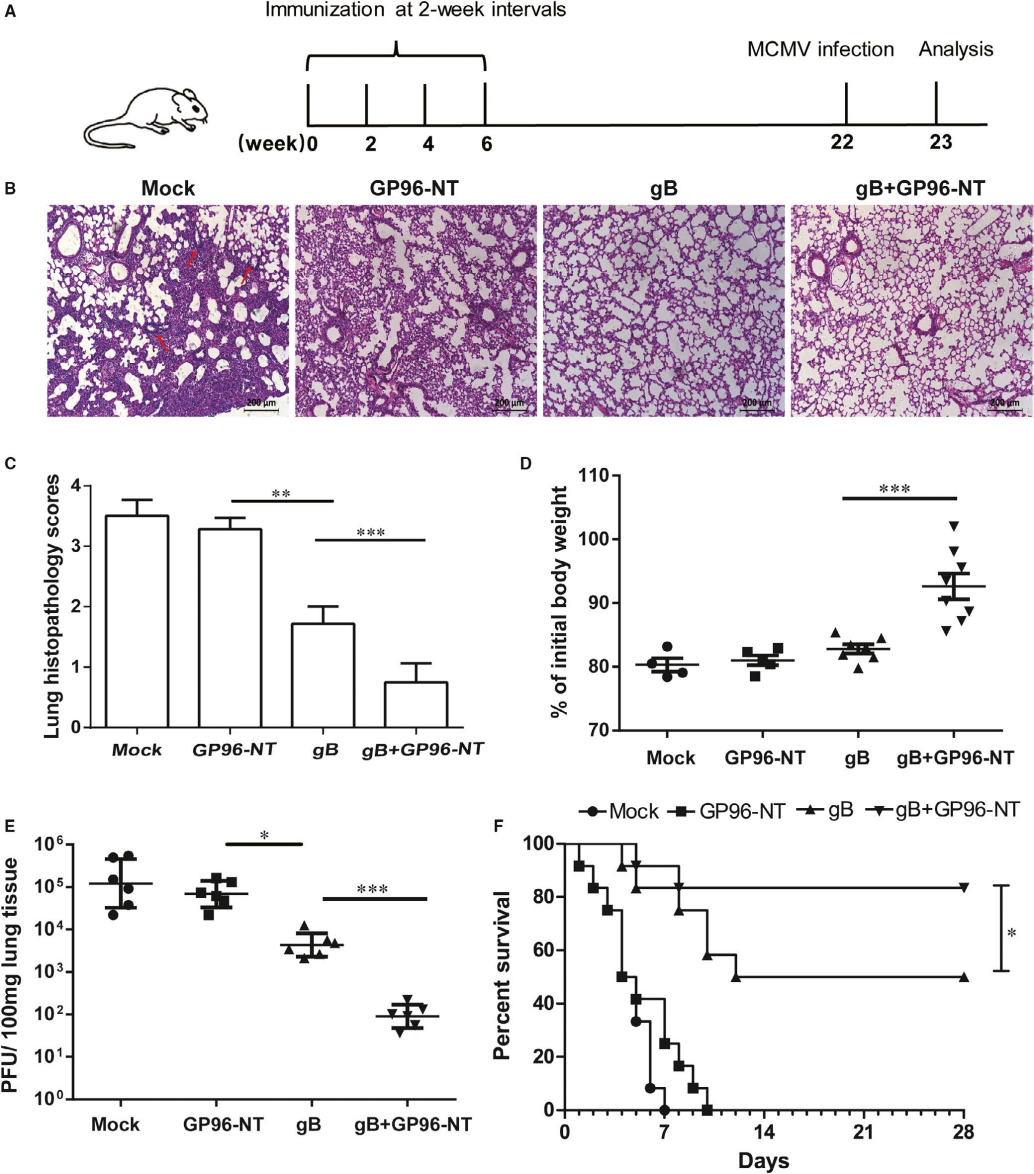
Schematic diagram of experimental protocol and the protective effect against MCMV pneumonitis by co‐immunization of PEI‐pgB and PEI‐pGP96‐NT. 7 days after 3LD_50_ MCMV challenge at week 16 after the last immunization, the protect effects were evaluated in mice. A, Schematic diagram. B, The representative lung section was shown for each group (magnification:100×). C, Pulmonary histopathological scores. D, Bodyweight loss. E, Viral titres in the lung tissues were measured by plaque assays. Data are from one representative experiment of 3 performed and presented as the mean ± SEM (n = 4‐8), and t test was used, *P < .05, **P < .01, ***P < .001. F, The survival rate of mice was observed until day 28 following a lethal dose of MCMV (5LD_50_) infection. Data are from one representative experiment of 3 performed (n = 12), Kaplan‐Meier analysis with log‐rank test was used, *P < .05

To answer whether co‐immunization of pgB and pGP96‐NT could provide an effective long‐lasting protection against MCMV pneumonitis, mice were *i.n*. treated with plasmids individually or in combination every 14 days for 4 times. 16 weeks after the last immunization, mice were challenged with a normal lethal dose (3LD_50_, 40 μl/mouse) of MCMV via the intranasal route for the induction of MCMV pneumonitis (Figure 3A). 7 days post‐infection, the development of pneumonitis was evaluated. HE‐stained lung sections showed the slightest lung inflammation and necrosis (red arrow) were observed in pgB/pGP96‐NT co‐vaccination group (Figure 3B). Using a previously reported standardized scoring system,^25^ it was found that although both co‐vaccination and pgB alone treated mice had significantly decreased pneumonitis scores compared with those of mock or pGP96‐NT‐treated mice, the co‐vaccination group had a significant decreased score than pgB alone group (*P* < .001; Figure 3C), indicating that pgB vaccination alone could offer partial protection against MCMV challenge, whereas co‐vaccination group further enhanced the pulmonary protection. Furthermore, weight loss was greatly improved in co‐vaccination‐treated mice (Figure 3D), indicating an alleviated viral infection. Indeed, viral burdens were remarkable decreased in co‐vaccination‐treated group than those from pgB vaccination‐treated group, although pgB vaccination alone could also partially decrease the viral burdens (Figure 3E). Finally, mice were administered with a lethal dose (5LD_50_) of MCMV and mouse survival was monitored daily up to 28 days. Figure 3F showed that all mice from mock immunized group died within 7 days upon MCMV challenge; meanwhile, about 50% of the mice from pgB immunization group died upon the lethal infection. However, nearly 83% of the mice from co‐vaccination group survived until 28 days, indicating that co‐vaccination of pgB and pGP96‐NT significantly enhanced protection against MCMV infection. Taken together, these data revealed that intranasal co‐immunization of pgB/pGP96‐NT provided a robust long‐lasting protection against MCMV pneumonitis.

### 3.4 Increased with pulmonary but not splenic CD8 T cells by pgB/pGP96‐NT with *i. n*. co‐vaccination

**Figure 4:**
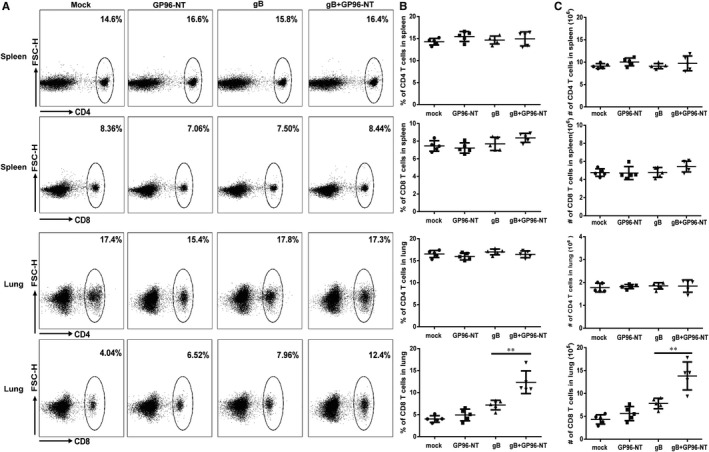
Pulmonary and splenic T‐cell responses against MCMV challenge by co‐immunization of PEI‐pgB and PEI‐pGP96‐NT. 7 days after 3LD_50_ MCMV challenge at week 16 after the last immunization, T‐cell responses were evaluated in mice. A, The representative flow cytometry profile of CD4 and CD8 T‐cell responses in spleen and lung upon MCMV challenge. Plots were pregated on CD45 cells. B, Frequency of CD4 and CD8 T cells in lung and spleen upon MCMV challenge. C, Number of CD4 and CD8 T cells in lung and spleen upon MCMV challenge. Data are from one representative experiment of 3 performed and presented as the mean ± SD (n = 5), t test was used, **P < .01

Functional T lymphocyte immunity—both in lymphoid organ and in non‐lymphoid tissue—was shown to substantially contribute to antiviral immunity.^28^ T cells are long known as the predominant effector cell type that controls acute pulmonary MCMV infection in related experimental mouse models.^29^, ^30^ It was also noted that intranasal MCMV infection could result in robust viral replication in lung and elicit a T‐cell responses with an EM phenotype.^31^ To test whether our immune route could elicit T‐cell responses and discriminate the contribution of lymphoid organ or non‐lymphoid organ T‐cell responses elicited by intranasal immunization to protect against MCMV infection, splenic and pulmonary T cells were determined 7 days after MCMV infection. Interestingly, although CD4 T cells in spleen and lung have no difference among 4 vaccination groups, the pulmonary but not splenic CD8 T cells had greatly increased in the co‐vaccination group than those in pgB immunization alone group (Figure 4). The T‐cell immune responses in all other 3 groups but not co‐immunization group demonstrated almost comparable to the primary T‐cell responses by *i.n*. MCMV infection described in other reports.^31^, ^32^ As shown in Figure 4B,C, the per cent and the absolute number of pulmonary CD8 T cells in the co‐vaccination group were 12.35 ± 2.58% and 13.82 × 10^5^ ± 3.06 × 10^5^, respectively, while those in pgB vaccination alone group were 7.16 ± 1.09% and 7.79 × 10^5^ ± 1.15 × 10^5^, respectively. The data suggested that pgB/pGP96‐NT co‐vaccination mice might have an association with increased pulmonary CD8 T cells that mediated a long‐term protection against MCMV challenge.

### 3.5 pgB/pGP96‐NT co‐immunization augments pulmonary‐resident CD8 T‐cell immune response

**Figure 5:**
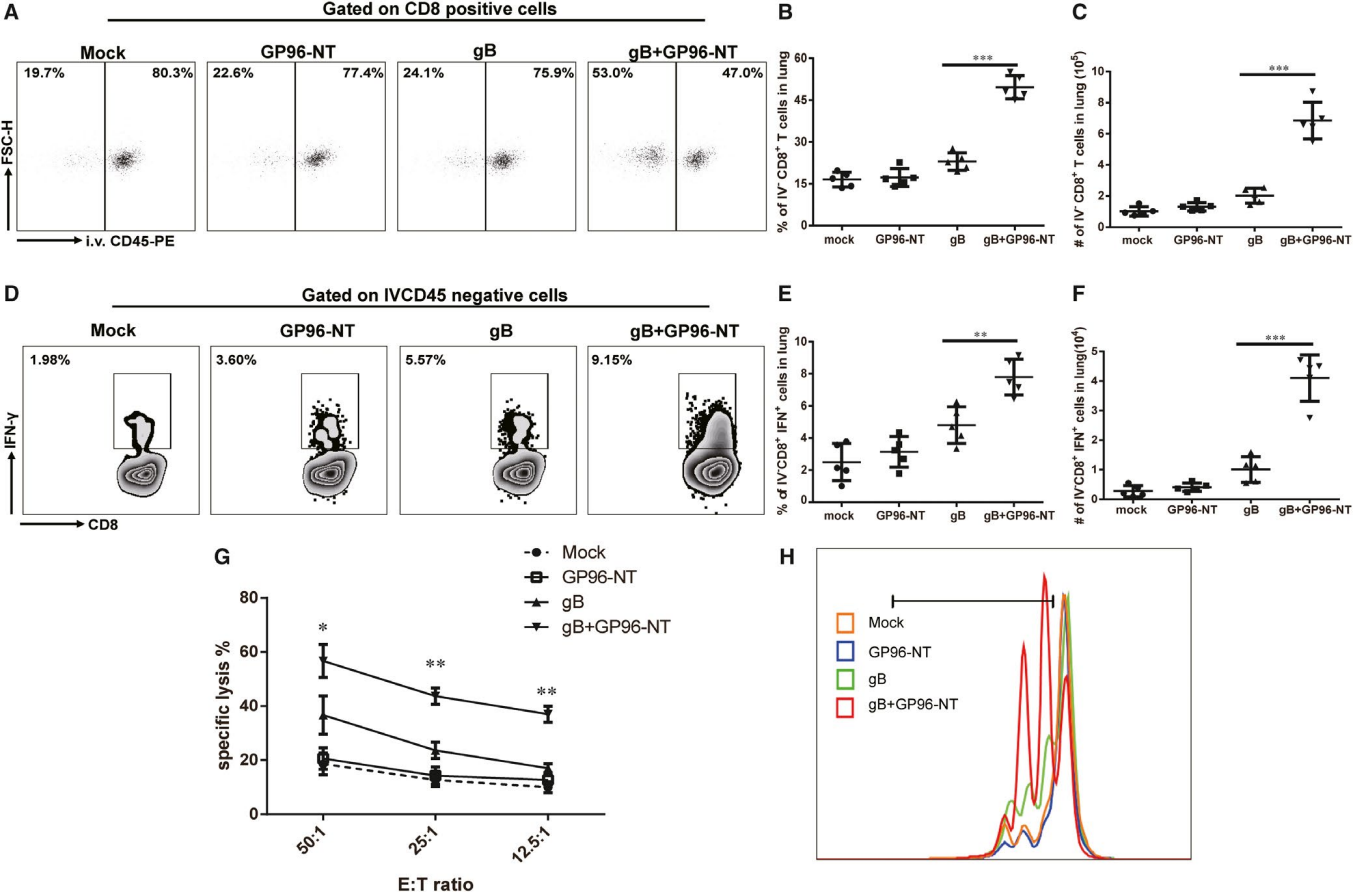
Pulmonary‐resident CD8 T‐cell responses against MCMV challenge by co‐immunization of PEI‐pgB and PEI‐pGP96‐NT. 7 days after 3LD_50_ MCMV challenge at week 16 after the last immunization, CD45‐PE antibody *iv* was administered 3‐5 min before mouse killing and lung CD8 T cells were analysed in mice. A, The representative flow cytometry profile of IVCD45^‐^CD8^+^ T cells responses in lung upon MCMV challenge. Plots were pregated on CD8 T cells. B, Frequency of IVCD45^‐^CD8^+^ T cells in lung. C, Number of IVCD45^‐^CD8^+^ T cells in lung. D, The representative flow cytometry profile of IVCD45^‐^CD8^+^ IFN‐γ^+^ T cells secreted in lung upon MCMV challenge. Plots were pregated on IVCD45^‐^ cells. E, Frequency of IVCD45^‐^ CD8^+^ IFN‐γ^+^ secreting T cells in lung. F, Number of IVCD45^‐^ CD8^+^ IFN‐γ^+^ secreting T cells in lung. At week 16 after the last immunization, lung IVCD45^‐^ CD8^+^ cells from immunized mice were sorted and stimulated in vitro. G, Cytotoxic T lymphocyte (CTL) activity in sorted lung IVCD45^‐^ CD8^+^ T cells. H, The lung IVCD45^‐^ CD8^+^ T lymphocyte proliferation assay was performed by CFSE labelling. Individual experiments were conducted thrice; results from one representative experiment are shown for each group of mice. Data are means ± SD (n = 5), and t test was used, *P < .05,**P < .01, ***P < .001

In non‐lymphoid tissue, it has been well documented that besides circulating T cells, non‐circulating tissue‐resident T cells also play an vital role against infection.^33^ In our setting, pulmonary CD8 T cells had been greatly enhanced in co‐vaccination group. In order to clarify whether the circulating CD8 T cells or resident CD8 T cells were the major source of pulmonary CD8 T‐cell increments. 16 weeks after the last immunization, mice challenged with MCMV and at day 7 post‐infection were *iv* injected with anti‐CD45‐PE antibody before killing. Circulating cells become labelled with the CD45^+^, whereas the Ab could not penetrate the tissue to stain the lung‐resident cells and would therefore remain unstained.^34^, ^35^ To our surprise, pulmonary IVCD45^‐^ CD8^+^ (resident) T cells rather than IVCD45^+^ CD8^+^ (circulating) T cells had remarkable increased in pgB/ pGP96‐NT co‐vaccination group compared to those in pgB immunization group (Figure 5A), the per cent and the absolute number of pulmonary IVCD45^‐^ CD8^+^ (resident) T cells in the co‐vaccination group were 49.64 ± 4.18% and 6.85 × 10^5^ ± 1.18 × 10^5^, respectively, while those in pgB vaccination alone group were 19.14 ± 3.13% and 2.02 × 10^5^ ± 0.48 × 10^5^, respectively (Figure 5B,C). Although the per cent of pulmonary IVCD45^+^ CD8^+^ (circulating) T cells was significantly decreased in co‐vaccination group compared with those in gB alone treatment group, the absolute number of pulmonary IVCD45^+^ CD8^+^ (circulating) T cells was comparable between co‐vaccination and gB alone treatment groups (Figure 5B,C). Taken together, these data indicated that pulmonary‐resident CD8 T cell rather than circulating CD8 T cells was the major source of pulmonary CD8 T‐cell increments.

CD8 T cells are known to produce effector cytokines, such as IFN‐γ to exert its antiviral immune response. Next, we assessed the secretion of IFN‐γ in IVCD45^‐^CD8^+^ T cells of mice that challenged with MCMV 16 weeks after the last immunization. At day 7 post‐infection, we found that IVCD45^‐^CD8^+^ T cells in pgB/pGP96‐NT co‐immunization group showed up‐regulated IFN‐γ secretion compared to that in pgB‐immunized group (Figure 5D), from 4.08 ± 1.14%, 1.00 × 10^4^ ± 0.44 × 10^4^ to 7.80 ± 1.13%, 4.10 × 10^4^ ± 0.79 × 10^4^ as shown in Figure 5E,F. In accordance, MCMV‐specific CTL activity was dramatically increased in pgB/pGP96‐NT co‐immunization group (Figure 5G). Compared with pgB‐immunized group, IVCD45^‐^CD8^+^ T cells showed more robust proliferation rate in pgB/pGP96‐NT co‐immunized mice (Figure 5H). Taken together, these data suggested that pgB/pGP96‐NT co‐vaccination induced a robust pulmonary‐resident specific T‐cell immunity and might protect against MCMV challenge even after 16 weeks of immunization.

### 3.6 pgB/pGP96‐NT enhances the induction of pulmonary memory‐resident CD8 T(CD8 T_RM_) cells

**Figure 6:**
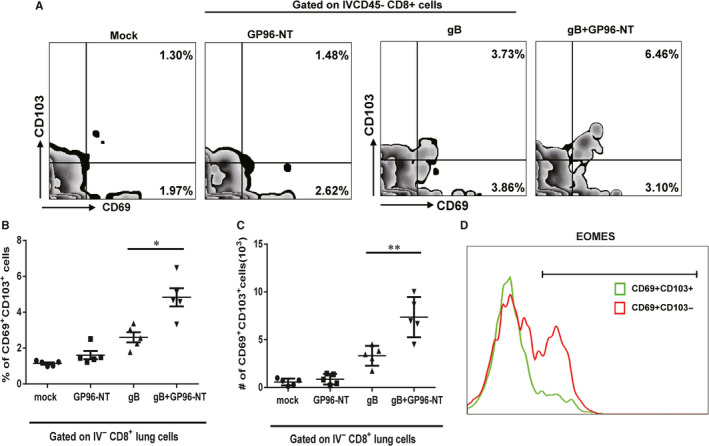
Pulmonary‐resident memory CD8 T cells induce by co‐immunization of PEI‐pgB and PEI‐pGP96‐NT. At week 16 after the last immunization, CD45‐PE antibody *iv* was administered 3‐5 min before mouse killing and lung CD8 T cells were analysed in mice. A, The representative flow cytometry profile of CD69^+^ CD103^+^ cells in lung. B, C, Frequency and number of CD69^+^ CD103^+^ cells in lung. D, The representative flow cytometry profile of Eomes expression in lung CD69^+^ CD103^+^ cells. Plots were pregated on IVCD45^‐^ CD8^+^ cells. Data are from one representative experiment of 3 performed and presented as the mean ± SD (n = 5), and t test was used, *P < .05, **P < .01

CD8 T_RM_ is a specific CD8 memory T‐cell subsets located in different non‐lymphoid tissues, such as liver, lung and gut.^36^, ^37^ These cells reside within the tissues and could not recirculate into the peripheral. Furthermore, CD8 T_RM_ differs a lot from conventional CD8 memory T cells regarding the phenotype and transcriptional profile.^38^ CD8 T_RM_ is well characterized by the expression of CD69 and CD103 on the cell surface. We assumed that pulmonary‐resident CD8 T‐cell increments in pgB/pGP96‐NT co‐immunized mice upon MCMV challenge might be due to enhancement of CD8 T_RM_ that facilitated resident CD8 T‐cell expansion after 4 times immunization. To test this hypothesis, 16 weeks after the last immunization, mice without MCMV challenge were *iv* administered with anti‐CD45‐PE antibody before killing to discriminate circulating (CD45^+^) and resident (CD45^−^) cells. The pulmonary CD8 T cells were then harvested and stained with CD69 and CD103. IVCD45^‐^ CD8 T_RM_ (CD69^+^CD103^+^) cells were enhanced in the lung (Figure 6A), and the proportion and number of this population was significantly higher in pgB/pGP96‐NT co‐immunized mice compared to those in pgB‐immunized mice (Figure 6B,C). However, CD69 and CD103 were not enough to identify CD8 T_RM_ cells, and CD8 T_RM_ cells were also known to express lower level of eomesodermin (Eomes) compared to classic T cells.^27^ We further validated our finding by detecting Eomes expression in CD69^+^CD103^+^ and CD69^+^CD103^−^ subsets. As expected, the pulmonary CD69^+^CD103^+^CD8^+^ T cells showed less Eomes expression compared to the pulmonary CD69^+^CD103^−^CD8^+^ T cells (Figure 6D). Hence, the data suggested that pgB/pGP96‐NT co‐immunization could increase CD8 T_RM_ cells that facilitated resident CD8 T‐cell expansion upon MCMV challenge, thereafter, provided a long‐lasting protection against MCMV infection.

### 3.7 Depletion of pulmonary CD8 T cells exacerbates MCMV pneumonitis

**Figure 7:**
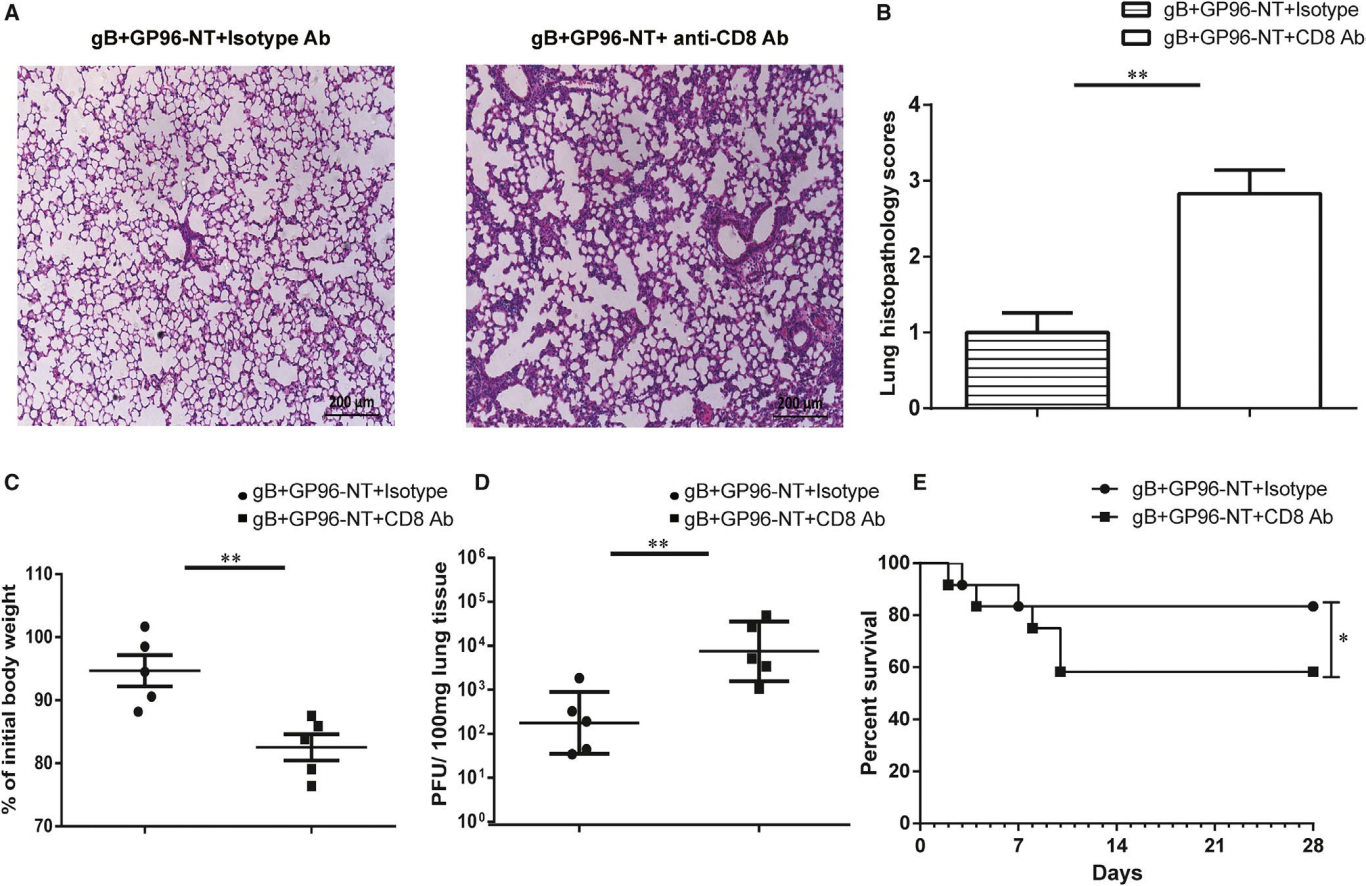
Pulmonary CD8 T cells are associated with the protective efficacy of PEI‐pgB and PEI‐pGP96‐NT vaccine. At week 16 after the last immunization, mice were *i.n*. injected with anti‐CD8 or isotype antibodies at −3 day and −1 day following MCMV infection. 7 days after MCMV challenge, the protection efficacy was evaluated in recipient mice. A, The representative lung section was shown for 2 groups (magnification:100×). B, Pulmonary histopathological scores. C, Bodyweight loss. D, Viral titres in the lung tissues were measured by plaque assays. Data are from one representative experiment of 3 performed and presented as the mean ± SD (n = 5), and t test was used,**P < .01. E, The survival rate of mice was observed until day 28 following a lethal dose of MCMV (5LD_50_) infection. Data are from one representative experiment of 3 performed (n = 12), and Kaplan‐Meier analysis with log‐rank test was used, *P < .05

Our data above suggested that our vaccination strategy provided a long‐lasting protection against MCMV pneumonitis by eliciting pulmonary‐resident CD8 T‐cell immune response. To further confirm that the long‐lasting protection depends on lung‐resident CD8 T cells, in pgB/pGP96‐NT co‐immunization group, 16 weeks after the last immunization, we performed site‐specific depletion of total pulmonary mucosal CD8 T cells by *i.n*. administration with αCD8 Ab or isotype control Ab 3 days and 1 day prior to MCMV challenge as previously reported^26^, ^27^ (depletion efficiency was shown in Figure S1). 7 days post‐infection, the severity of pneumonitis, weight loss and viral burdens were evaluated. Histological analysis showed that more severe pulmonary inflammation was observed in pulmonary CD8 T‐cell depletion group than that in isotype‐treated controls (Figure 7A,B). Furthermore, weight loss was greatly decreased in depletion group (Figure 7C). In accordance, viral burdens were remarkable increased in depletion group (Figure 7D). Finally, mice were also treated with a lethal MCMV (5LD_50_), and the survive rate was observed. The results showed that after pulmonary CD8 T‐cell depletion, pgB/pGP96‐NT‐immunized mice showed a poorer survival rate compared to that of control group (Figure 7E). In conclusion, these data suggested that our vaccination strategy might provide a long‐term protection against MCMV infection by boosting pulmonary‐resident CD8 T cells.

## 4 DISCUSSION

Human cytomegalovirus is a pathogenic virus which causes pneumonitis, retinitis, hepatitis and encephalitis in human.^39^ Among them, CMV‐induced pneumonitis had been recognized as a major concern due to its high mortality, high chance of occurrence and lack of effective antiviral therapy.^40^ Because of its strict species specificity, it was hard to establish an animal model of HCMV infection. However, both HCMV and MCMV showed tropism to similar host cells and also lead to comparable histopathology in the site of infection.^2^ Moreover, as early as in 1978&1982, Jordan and Shanley had described an interstitial pneumonitis caused by MCMV after intranasal application of the virus.^41^, ^42^ Thus, MCMV‐induced murine pneumonitis could be used in the development of vaccine against HCMV pneumonitis. DNA vaccination was a promising approach to achieve long‐term humoral and cellular immune responses in multipole animal disease model^43^ and was closely associated with protection against pneumonitis.^44^ Nevertheless, the appropriate vaccination strategy and the potent adjuvants were needed for success of DNA vaccine in inducing a long‐term immune response.^43^ Our present study clearly revealed that co‐vaccination of pgB/pGP96‐NT intranasally could elicit a long‐lasting protection against MCMV pneumonitis and suggested that pulmonary‐resident CD8 T cells might be responsible for the long‐term protection.

Glycoprotein B, which was involved in the viral entry into almost all types of host cells, served as a promising target for inclusion in a human vaccine. As a virion fusion protein for herpesviruses including CMV,^45^ gB had been widely used in the development of experimental vaccination strategies. Mucosal immunization with a replication‐deficient adenovirus vector expressing MCMV glycoprotein B induced mucosal and systemic immunity.^46^ Additionally, several studies showed that congenital infection and mortality in pups were reduced following gB DNA or recombinant protein subunit immunization.^47^, ^48^ Finally, gB‐based vaccine showed an efficacy of approximately 50% protection.^7^ These vaccination studies, however, mainly centred on the gB antibody responses while gB‐mediated cellular immune responses were not fully interpreted. Our study demonstrated that although pgB/pGP96‐NT co‐immunization could induce humoral immune responses (Figure 2C), it could induce pulmonary CD8 T_RM_ cell responses (Figure 6A) that facilitated pulmonary‐resident CD8 T‐cell expansion upon MCMV challenge (Figure 5A), which might provide new avenues to enhance protection efficacy upon MCMV pneumonitis.

The heat‐shock protein (HSP) is a family of molecular chaperones located in the endoplasmic reticulum. Among several HSPs, GP96 had been widely used as immune adjuvant to boost the immune‐promoting responses induced by vaccination.^49^ The characteristics of GP96 included its capability to bind to antigenic peptides, cross‐presentation of associated peptides and activation of peptide‐specific CTL responses. It was reported that GP96 or its N‐terminal domain as an adjuvant could elicit specific T immunity in HCV infection models.^24^, ^50^ Förster group had reported that T cells had to be present in nodular inflammatory foci (NIFs) of lung in high numbers to efficiently control MCMV spread and IFN‐γ had the potential to reduce plaque growth on primary lung stromal cells.^51^ Consistent with previous reports, our data showed that pGP96‐NT plasmid as an adjuvant could enhance the frequency and absolute number of pulmonary‐resident CD8 T cells (Figure 5B,C), and also enhanced the frequency of IFN‐γ producers in co‐vaccination mice compared to that in the pgB‐immunized mice(Figure 5D).

In this study, we successfully confirmed that pgB and pGP96‐NT plasmids could express in vitro and in vivo, respectively (Figure 1). Subsequently, to estimate the quality and quantity of immune responses induced by vaccines, we employed pgB and/or pGP96‐NT plasmid to immunize Balb/c mice. Our data showed that humoral and cellular immune responses were strongly elicited. SIgA, sera IgG and antiviral‐related cytokine levels robustly induced by pgB/pGP96‐NT or pgB vaccines (Figure 2C,D), however, pulmonary but not splenic CD8 T cells had significantly increased by pgB/pGP96‐NT vaccines than that by other 3 group vaccines (Figure 2B), suggesting in addition to protection by humoral immunity, CD8 T cells might contribute to additionally protect against MCMV challenge. Nevertheless, CD4 T cells showed no change in both lung and spleen by gB/GP96‐NT or gB alone vaccines (Figure 2A).

In our setting, we next investigated whether our vaccine design strategy could provide a long‐term protection upon MCMV challenge. Compared within the 4 vaccination groups that were tested, pgB/pGP96‐NT co‐immunization‐treated group greatly ameliorated MCMV pneumonitis 16 weeks after the last vaccination (Figure 3), which was in line with our hypothesis. Besides, both lung histopathology and viral burdens were also partially alleviated by pgB alone vaccination (Figure 3C,E); however, co‐immunization group further enhanced the protection, indicating that (a) pgB alone consistent with Huang's finding^9^ could partially offer protection over 4 times immunization; (b) in addition to protective effects of known mechanisms such as humoral immune responses by pgB immunization,^4^ it seems that there might be alternative mechanisms to play a protective role in controlling of pulmonary MCMV infection by pgB/pGP96‐NT co‐immunization.

CD8 T cells play a vital in eliminating virus‐infected cells. In a MCMV infection mouse model, in vivo depletion of CD8 T cells had a stronger effect on lung virus loads than depletion of CD4 T cells only.^30^ Reddehase^52^ and Cicin‐Sain^53^ groups also indicated that CD8 T cells and tissue‐resident CD8 T cells had a great role in control of MCMV infection. Past vaccination strategies had been shown to induce a potent long‐term protection against MCMV challenge through strong CD8 T‐cell responses.^54^ Moreover, in 2016, Oduro described that CMV infection via the intranasal route offered a robust model of immunity upon mucosal CMV infection, which intranasal infection induced robust and long‐term virus replication in the lungs but limited replication in the spleen.^31^ Reuter also reported that in an OVA‐induced asthma model, intratracheal MCMV infection and OVA sensitization combined induced pulmonary CD8 T‐cell responses.^32^ Thus, we decided to explore whether CD8 T cells at least also had a protective role in our vaccine design for defence MCMV challenge. Interestingly, our data showed that the CD8 T‐cell responses in all groups except the co‐immunization group were almost comparable to the primary response described in Oduro and Reuter reported^31^, ^32^; however, pulmonary but not splenic CD8 T cells were drastically enhanced in pgB/pGP96‐NT group compared with that in other groups (Figure 4).

During viral infection, T cells might be quickly converted into large numbers of effector T cells that home to specific inflammation site to fight against pathogens, which might result in pulmonary CD8 T‐cell increment in our setting. To address this question, we had applied *iv* injection of PE labelled anti‐CD45 antibody before sampling, which made it possible to discrimination of tissue‐resident leucocytes from circulating leucocytes.^34^, ^35^ Although in Figure 3B showed that the occurrence of lung necrosis by MCMV infection might result in the risk of vascular or endothelial barrier leakage, which might compromise the accuracy of CD45 staining, the proven method was applied in several different models. In an acute pulmonary acid injury model which pulmonary necrosis might occur, Evans blue dye extravasation assay confirmed that circulating leucocytes primarily resided in the vascular wall of the lung.^55^ Tumour cells are often in an hypoxic condition that causes tumour tissue inflammation and necrosis. In a renal carcinoma model, collagen type IV staining for glomerular basement membrane surrounds endothelial vessels also confirmed that *iv*‐injected Ab staining was limited to vascular‐localized site and did not infiltrate the tumour.^34^ Additionally, Ab circulation time was strictly limited in order to ensure the capillary bed did not be permitted perivascular leakage of Abs since Abs was allowed to circulate in blood vessel only 3‐5 minutes prior to killing and lung harvest within 12 minutes in order to prevent Abs leakage. Therefore, CD45 *iv* staining might be an appropriate way to discriminate between circulating and resident CD8 T cells. To our surprise, data revealed a statistically significant increment of pulmonary‐resident CD8 T cells was observed while the circulated CD8 T cells remained relatively unchanged (Figure 5), indicating local site rather than systemic CD8 T‐cell immune responses might be serve as first‐line defender against the subsequent infection.

During the past decade, a specific memory CD8 T‐cell subset, known as CD8 T_RM_, has emerged as an important guardian to protect against pathogens at sites of previous infection.^56^ More potent vaccine efforts were based on induction and maintenance of this non‐circulating, resident T‐cell subset. Moreover, in vivo studies had shown that MCMV resulted in the formation of CD8 T_RM_ cells.^13^, ^57^, ^58^, ^59^ Therefore, we hypothesized that, at 16 weeks after the last co‐immunization, the expansion of pulmonary‐resident CD8 T cells upon MCMV challenge might be due to CD8 T_RM_ cells enhanced. In accordance with our hypothesis, data reflected that increased CD69^+^CD103^+^ CD8 T‐cell population in lung (Figure 6A), which might be converted into pulmonary‐resident effector CD8 T cells that exhibited CTL activity (Figure 5G). Site‐specific anti‐CD8 antibody administration depleted CD8 T_RM_ which aggravated influenza challenge by a MCMV‐based vaccine.^27^ The majority (>90%) of the Ag‐specific T cells would die 4 months after the last immunization, while the rest would differentiate into memory T cells.^60^ Thus, we performed site‐specific depletion of pulmonary CD8 T cells to investigate whether the protection in the co‐vaccination mice was dependent on pulmonary CD8 T_RM_ cells. Indeed, we found that pulmonary CD8 T cell depletion abrogated prevention of MCMV infection (Figure 7). Although other cells such as B cells in the lung might also contribute to protection, it did not neglect that our observation on the elicitation of CD8 T_RM_ cells in lung by co‐vaccination. However, whether the increments of CD69^+^CD103^+^ CD8 T_RM_ cells in lung induced by co‐immunization were specific for MCMV or not, it should be further identified with an antigen‐specific tetramer staining.

In conclusion, our study showed that the use of heat‐shock protein N‐terminal domain of GP96 as an adjuvant could efficiently enhance the immunogenicity of MCMV gB DNA vaccine and mucosal resident memory CD8 T‐cell immune responses elicited by gB/GP96‐NT co‐immunization offered a long‐term protection against MCMV pneumonitis. Our results might unlock an novel vaccination strategy for MCMV pneumonitis.

## CONFLICT OF INTEREST

None.

## AUTHOR CONTRIBUTION

**Bingnan Guo:** Conceptualization (lead); Data curation (lead); Formal analysis (lead); Funding acquisition (lead); Investigation (lead); Methodology (lead); Project administration (lead); Resources (lead); Validation (lead); Writing‐original draft (lead); Writing‐review & editing (lead). **Peifeng Xu:** Formal analysis (supporting); Investigation (supporting); Writing‐original draft (supporting). **Dafei Chai:** Formal analysis (supporting); Funding acquisition (supporting); Writing‐original draft (supporting); Writing‐review & editing (supporting). **Lei Cao:** Methodology (supporting); Validation (supporting). **Lin Liu:** Formal analysis (supporting); Writing‐original draft (supporting); Writing‐review & editing (supporting). **Tengfei Song:** Writing‐original draft (supporting). **Shuqun Hu:** Methodology (supporting). **Yuling Chen:** Resources (supporting); Supervision (supporting). **Xianliang Yan:** Conceptualization (lead); Resources (lead); Supervision (lead); Writing‐original draft (lead); Writing‐review & editing (lead). **Tie Xu:** Conceptualization (lead); Funding acquisition (lead); Resources (lead); Supervision (lead); Writing‐original draft (lead); Writing‐review & editing (lead).

## Supporting information

Fig S1
