# Supplementary figures and images for: gB co‐immunization with GP96 enhances pulmonary‐resident CD8 T cells and exerts a long‐term defence against MCMV pneumonitis
Source: J Cell Mol Med. 2020 Nov 6;24(24):14426–40. doi: 10.1111/jcmm.16065 (PMC7754068; doi:10.1111/jcmm.16065)

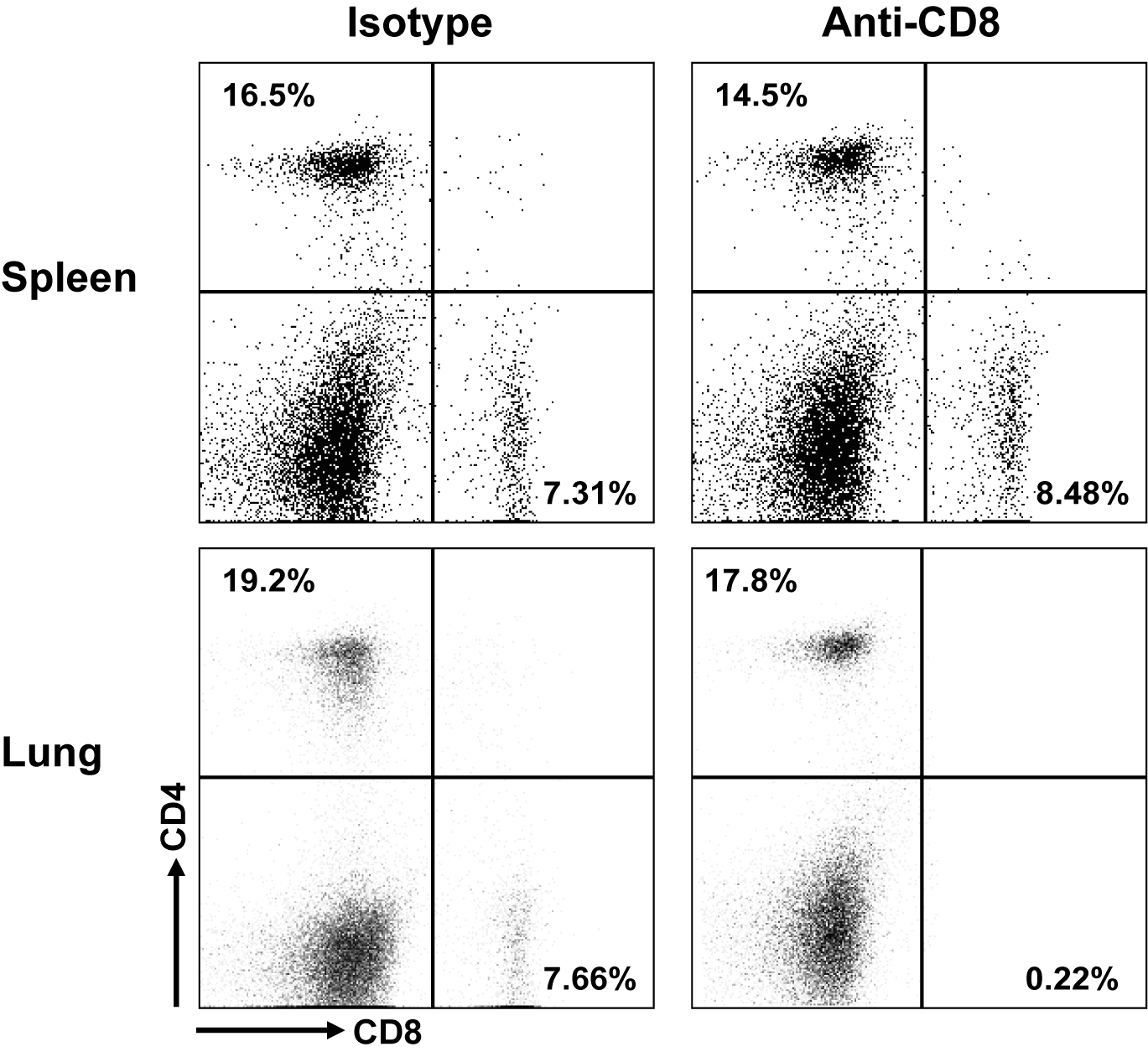

Supplement: Supplementary file 1 — Fig S1 [file JCMM-24-14426-s001.tif]
